# Bone fractures and feeling at risk for osteoporosis among women in Japan: patient characteristics and outcomes in the National Health and Wellness Survey

**DOI:** 10.1007/s11657-014-0199-7

**Published:** 2014-11-13

**Authors:** Masayo Sato, Jeffrey Vietri, Jennifer A. Flynn, Saeko Fujiwara

**Affiliations:** 1 Eli Lilly K.K., Lilly Research Laboratories, Kobe, Japan; 2 Kantar Health, Health Outcomes Practice, Via Paleocapa 7, 20121 Milan (MI), Italy; 3 Hiroshima Atomic Bomb Casualty Council, Hiroshima, Japan

**Keywords:** Osteoporosis, Fractures, Risk perception, Quality of life, Japan

## Abstract

**Summary:**

Women aged 50 and older in Japan were compared according to perceived risk for osteoporosis and fracture history. Perceived risk was associated with family history of osteoporosis but few other risk factors. Few felt at risk, and perception was only loosely related to epidemiological risks, indicating a need for patient education.

**Purpose:**

Osteoporosis is prevalent but underdiagnosed and undertreated. This study was conducted to explore characteristics associated with history of fractures and feeling at risk for osteoporosis in women aged 50 and older in Japan.

**Methods:**

Data were provided by a large annual survey representative of Japanese aged 18 and older. Women 50 and older without diagnosed osteoporosis were categorized into four mutually exclusive groups based on fracture history since age 50 and feeling at risk for developing osteoporosis. Sociodemographic and health characteristics were compared across groups using bivariate statistics, and health outcomes were compared using generalized linear models.

**Results:**

A total of 16,801 women aged 50 and older were included in the analyses. Most (*n* = 12,798; 76.2 %) had no fracture since age 50 and did not feel at risk for osteoporosis, 12.9 % (*n* = 2170) felt at risk but had no fracture, 8.7 % (*n* = 1455) did not feel at risk despite having a fracture, and 2.2 % (*n* = 378) had a fracture and felt at risk for osteoporosis. Feeling at risk was slightly more common among those with than without a fracture since age 50 (20.6 vs. 14.5 %, *p* < 0.001). Feeling at risk was most associated with family history of osteoporosis, though known risk factors for fracture did not significantly differ across the fracture/perceived-risk group.

**Conclusions:**

Approximately 15 % of women in Japan aged 50 and older felt at risk for developing osteoporosis in the future, far fewer than expected by epidemiologists. Risk perception was only loosely related to epidemiological risks for fracture, indicating a need for patient education.

## Introduction

Osteoporosis is a major public health issue in Japan, though not always recognized as such [1]. It is estimated up to a quarter of women of all ages in Japan have osteoporosis, with prevalence rising sharply after age 50 [2]. Despite the high prevalence, the condition is believed to be underdiagnosed and undertreated [3]. Fractures caused by osteoporosis contribute to back pain, reduce quality of life, and interfere with activities of daily living.

The consequences of osteoporosis also impose an economic burden on society, with costs of hip and vertebral fractures estimated at approximately 8.0 and 9.9 billion yen (US$78 and 97 million), respectively [4], with costs increasing with the age of the patient [5]. Recently the cost per hip fracture in Japan was identified as among the highest in the Asia-Pacific region, with average hospital cost reported as US$27,599 and involving an average of 38 hospital days [6]. While the incidence of hip fractures has stabilized in the West, the incidence of these fractures is increasing in Japan [7].

A variety of treatments have demonstrated effectiveness in slowing or halting bone loss and reducing fracture risk in osteoporosis [8, 9]. Though multiple treatment options are available in Japan, only a low proportion of individuals suffering osteoporotic fractures in Japan are treated prior to fracture, suggesting many of those most at risk for fracture are not being identified and treated until after a fracture occurs [10, 11]. Indeed, osteoporosis has been called a silent disease because bone loss typically occurs without symptoms, becoming apparent only after the individual sustains a fragility fracture—that is, a fracture resulting from a trauma that would not break a healthy bone, such as a mild fall from standing height—or the individual has a bone densitometry test. Lack of awareness among those at risk and the asymptomatic nature of the disease are both barriers to effective fracture prevention.

Because osteoporosis is an underrecognized and underdiagnosed condition, it is important to understand the population that has not been diagnosed with osteoporosis, whether they understand the condition, take steps to prevent it, or feel at risk of developing it. Likewise, it is important to ascertain to what extent perceived risk of developing osteoporosis and actual risk for developing the condition coincide, with those at most risk also being the most likely to perceive being at risk. Previous research in the US has demonstrated that individuals’ perception of their own risk for osteoporotic fractures is not closely related to established epidemiological risk factors [12], but this relationship has not been assessed among women in Japan.

Likewise, there is little information on women’s perceptions of osteoporosis in Japan, how widely women who are actually at risk feel at risk, or what is driving perceived risk. In particular, prior fracture is the greatest predictor of future fracture [13, 14], but it is not clear how strongly women take their own fracture history into account when assessing their risk for osteoporosis. The Fracture Risk Assessment Tool (FRAX) calculator developed to estimate 10-year risk for major osteoporotic fracture includes a number of other risk factors, including age, smoking, alcohol consumption, and use of long-term glucocorticoid medication [15], and the extent to which an individual’s perceived risk is sensitive to these epidemiological risk factors is also unknown. Bone mineral density (BMD) scanning is available as part of recommended osteoporosis screening in Japan, but it is not clear how widespread the practice is, and in 2005, fewer than 5 % of those eligible for screening participated [16].

The current study was conducted to better understand women in Japan age 50 years and older in terms of their perceived risk of osteoporosis and their experience with fractures and to assess the relationship between patient characteristics, perceived risk, and fracture history among women without diagnosed osteoporosis in this demographic group.

## Methods

### Data source

The current study used data from the 2008 (*N* = 20,000), 2009 (*N* = 20,573), 2010 (*N* = 25,000), and 2011 (*N* = 30,000) Japan National Health and Wellness Surveys (NHWS; Kantar Health, New York, NY), an annual, cross-sectional study of individuals aged 18 years or older in Japan. Response rates for these surveys were 40.0, 22.7, 24.9, and 15 % in 2008 through 2011, respectively. Because sampling for NHWS is without regard to previous participation, individuals can participate in multiple years of the survey, and approximately 10 % of the total responses (9206 of 95,573) was made by an individual who completed the survey in a subsequent year (e.g., completed the survey in 2008 and again in 2010). In these cases, only the most recent response made by the individual was included; older responses made by the same individual were excluded from analysis in order to avoid including the same respondent more than once. Only women aged 50 and older were included in the present study. The NHWS includes information related to diagnosis and treatment of a broad variety of conditions, health risk behaviors, and health-related outcome data. Potential respondents to the NHWS are recruited through an existing web-based consumer panel, which recruits its members through opt-in emails, co-registration with panel partners, e-newsletter campaigns, banner placements, and both internal and external affiliate networks. All panelists explicitly agreed to be a panel member, registered with the panel through a unique email address, and completed an in-depth demographic registration profile.

The sample for NHWS is selected from this panel using a stratified random sample framework with quotas based on gender and age. Previous research has found the demographic composition of the Japan NHWS to be comparable to that of the Japanese adult population on important parameters [17]. Though there were some minor changes and enlargements to the NHWS questionnaire during the years included in this study, the questions analyzed in the present study remained consistent, allowing for the combination of multiple years of survey data.

All respondents to NHWS provided informed consent, and the study was approved by Essex Institutional Review Board (Lebanon, NJ).

### Measures

All measures were by self-report.

#### Sociodemographic characteristics

Among the variables included in the NHWS, age, marital status, employment status, level of education, and household income were included in the present analyses.

#### General health characteristics

Current use of cigarettes, daily use of alcohol, and whether an individual had exercised vigorously in the past month were included. Body mass index (BMI) was calculated from reported height and weight.

#### Comorbid health conditions

The Charlson Comorbidity Index (CCI) [18] was used to summarize the overall comorbidity burden of the respondents. This index weights the presence of the following conditions and sums the result: HIV/AIDS, metastatic tumor, lymphoma, leukemia, any tumor, moderate/severe renal disease, hemiplegia, diabetes, mild liver disease, ulcer disease, connective tissue disease, chronic pulmonary disease, dementia, cerebrovascular disease, peripheral vascular disease, myocardial infarction, congestive heart failure, and diabetes with end organ damage. The greater the total index score, the greater the comorbid burden on the patient.

#### Perceived risk of developing osteoporosis

Respondents were compared on the basis of their perceived risk of developing osteoporosis in the future and whether they had experienced a fracture since age 50. Perceived risk was assessed with an item asking the respondent to indicate which of a variety of age-related conditions the respondent felt at risk of developing in the future, of which osteoporosis was one. Respondents who selected osteoporosis were considered to feel at risk of osteoporosis.

#### Fractures since age 50

Respondents were asked to indicate the number of bone fractures they had experienced since age 50. Those who indicated one or more fractures were considered to have had a fracture.

#### Fracture risks and preventative steps

Use of oral glucocorticoids was assessed by assessing the current medications the respondent reported in the survey; respondents also indicated whether they had completed menopause, if they had back pain, and if they had a family history of osteoporosis. Respondents were also asked if they were taking steps to prevent a variety of conditions, including osteoporosis and, if so, what specific steps they were taking. Respondents also indicated if they had ever had a bone mineral density scan.

#### Health status

All respondents completed the revised Medical Outcomes Study 12-Item Short Form Survey Instrument (SF-12v2), a multipurpose, generic instrument comprising 12 questions [19]. This instrument can be used to summarize functional health by two summary scores: the physical component summary (PCS) and mental component summary (MCS). Each score has a mean of 50 and a standard deviation of 10 for the US population, with higher scores indicating better health. Several of the items from the SF-12v2 can be used to generate a health state utility score, the SF-6D. The SF-6D is a preference-based single-index measure for health using general population values [20]. The SF-6D index has interval scoring properties and yields summary scores on a theoretical 0–1 scale (with an empirical floor of 0.3). Higher scores indicate better quality of life.

#### Work productivity and activity impairment

Impairment to work productivity was assessed using the Work Productivity and Activity Impairment (WPAI) questionnaire, a six-item validated instrument which consists of four metrics: absenteeism (the percentage of work time missed because of one’s health in the past 7 days), presenteeism (the percentage of impairment experienced while at work in the past 7 days because of one’s health), overall work productivity loss (an overall impairment estimate that is a combination of absenteeism and presenteeism), and activity impairment (the percentage of impairment in daily activities because of one’s health in the past 7 days) [21]. Only respondents who reported being full-time or part-time employed provided data for absenteeism, presenteeism, and overall work impairment. All respondents provided data for activity impairment.

#### Healthcare use

The number of physician visits (including visits to physicians, dentists, and nurses), the number of emergency room (ER) visits, and the number of times hospitalized in the past 6 months were used to define healthcare use.

### Analysis

The sample was characterized with descriptive statistics, and Spearman’s correlation was used to quantify the strength of the relationship between perceived risk and history of fracture. Women were categorized into four groups based on their perceived risk of osteoporosis and report of fractures since age 50: (1) not feeling at risk for osteoporosis, no fracture; (2) not feeling at risk for osteoporosis, with fracture; (3) feeling at risk for osteoporosis, no fracture; and (4) feeling at risk for osteoporosis, with fracture. These groups were first compared using one-way ANOVA for continuous variables and chi-square tests for categorical variables. The different perceived risk/fracture categories were compared using generalized linear models, with each outcome modeled separately. Models for MCS, PCS, and SF-6D incorporated a normal distribution and an identity link function and so were equivalent to linear regressions. For work productivity impairment, activity impairment, and healthcare use variables, models incorporated a negative binomial distribution with a log-link function to better accommodate the skewed nature of the data. All models were adjusted for age, university education (completed 4-year degree vs. less), smoking status (current vs. former vs. never), exercise (in the previous month vs. not), daily alcohol use (yes vs. no), household income (above median vs. below median vs. decline to answer), BMI category (underweight vs. normal weight vs. overweight vs. obese vs. decline to answer), marital status (never married, divorced, or separated vs. widowed vs. married/living with partner), and the CCI. The main predictor of interest was the perceived risk and fracture group, which was a dummy-coded variable where the group not feeling at risk and without fractures served as the reference category against which each of the other three categories was tested. Regression-adjusted means and standard errors were also calculated for each group to assist in interpretation.

Factors typically considered health outcomes may also be driving perceived risk of osteoporosis; the cross-sectional design of the current study does not allow us to assess whether risk preceded poor outcomes or poor outcomes increase the perception of risk. Therefore, a binary logistic regression was used to test the association between feeling at risk for developing osteoporosis and the set of demographics and outcomes that may plausibly precede feeling at risk for osteoporosis. Because taking steps to prevent osteoporosis seems particularly unlikely to lead to (rather than result from) feeling at risk for the condition, taking such steps was excluded from the analysis.

## Results

A total of 16,801 women without self-reported osteoporosis were included in the analysis. The respondents had a mean age of 60 years (range 50–93), 37 % were employed, and approximately 11 % indicated they were taking steps to prevent developing osteoporosis in the future. Forty-nine percent indicated they had never had a bone mineral density test. Approximately 11 % had fractured a bone since age 50, and approximately 15 % felt at risk for developing osteoporosis in the future.

Feeling at risk for developing osteoporosis in the future was more common among those who had experienced a fracture since age 50 than those who had not experienced a fracture in that time (20.6 vs. 14.5 %, *p* < 0.001), though the magnitude of the correlation between perceived risk and history of fracture was small (*r*_s_ = 0.05).

Most (*n* = 12,798; 76.2 %) had no fracture since age 50 and did not feel at risk for osteoporosis, 12.9 % (*n* = 2170) felt at risk but had no fracture, 8.7 % (*n* = 1455) did not feel at risk despite having a fracture, and 2.2 % (*n* = 378) had a fracture and felt at risk for osteoporosis. Respondent characteristics are compared among the four groups in Table 1. The size of the sample made the statistical tests sensitive to very small differences across the groups, and most variables were significantly different across the groups. The groups that had experienced fractures were several years older than the groups without fractures on average and so had more time to experience a fracture since age 50. The groups with fractures also had lower rates of employment and higher rates of menopause than the group with fractures.

**Table 1:** Demographic and health characteristics among women in Japan age 50 and older by perceived risk of osteoporosis and fracture history

	Not feeling at risk, no fractures (*N* = 12,798)		Not feeling at risk, fracture (*N* = 1455)		Feeling at risk, no fractures (*N* = 2170)		Feeling at risk, fractures (*N* = 378)		
	Mean	SD	Mean	SD	Mean	SD	Mean	SD	*p* value^a^
Age of the respondent	59.43	7.61	64.52	7.52	59.04	7.34	63.90	7.25	<0.0001
BMI	21.92	3.27	22.15	4.02	21.29	2.91	21.46	2.79	<0.0001
CCI	0.12	0.40	0.19	0.52	0.16	0.49	0.17	0.51	<0.0001
	*n*	%	*n*	%	*n*	%	*n*	%	*p* value^b^
What is your marital status?									<0.0001
Married/living with partner	9918	77.5 %	1040	71.5 %	1636	75.4 %	267	70.6 %	
Widowed	958	7.5 %	175	12.0 %	155	7.1 %	46	12.2 %	
Never married/divorced/other	1922	15.0 %	240	16.5 %	379	17.5 %	65	17.2 %	
Employed	4834	37.8 %	398	27.4 %	819	37.7 %	110	29.1 %	<0.0001
Completed 4-year college	3251	25.4 %	309	21.2 %	627	28.9 %	96	25.4 %	<0.0001
Annual household income									<0.0001
¥5,000,000 or above	6257	48.9 %	572	39.9 %	1171	54.0 %	172	45.5 %	
Less than ¥5,000,000	4800	37.5 %	673	46.3 %	834	38.4 %	169	44.7 %	
Decline to answer	1741	13.6 %	210	14.4 %	165	7.6 %	37	9.8 %	
BMI categories									<0.0001
Underweight	1251	9.8 %	175	12.0 %	321	14.8 %	45	11.9 %	
Normal	9248	72.3 %	1017	69.9 %	1599	73.7 %	287	75.9 %	
Overweight	1520	11.9 %	189	13.0 %	180	8.3 %	39	10.3 %	
Obese	205	1.6 %	31	2.1 %	24	1.1 %	2	0.5 %	
Decline to answer	574	4.5 %	43	3.0 %	46	2.1 %	5	1.3 %	
Currently smokes	1809	14.1 %	172	11.8 %	351	16.2 %	50	13.2 %	0.0028
Currently drinks	7737	60.5 %	786	54.0 %	1385	63.8 %	228	60.3 %	<0.0001
Daily alcohol use	1421	11.1 %	170	11.7 %	240	11.1 %	41	10.8 %	0.9191
Currently exercises	6010	47.0 %	767	52.7 %	1042	48.0 %	195	51.6 %	0.0002
Back pain	568	4.4 %	82	5.6 %	171	7.9 %	38	10.1 %	<0.0001
On glucocorticoids	261	2.0 %	36	2.5 %	67	3.1 %	12	3.2 %	0.0100
Completed menopause	5794	45.3 %	891	61.2 %	1026	47.3 %	244	64.6 %	<0.0001
Visited physician (in the prior 6 months)	8469	66.2 %	1165	80.1 %	1662	76.6 %	329	87.0 %	<0.0001
Visited ER (in the prior 6 months)	370	2.9 %	87	6.0 %	67	3.1 %	22	5.8 %	<0.0001
Visited hospital (in the prior 6 months)	450	3.5 %	109	7.5 %	77	3.5 %	25	6.6 %	<0.0001
Have you ever had a bone mass density test/scan?									<0.0001
Yes	5767	45.1 %	884	60.8 %	1172	54.0 %	249	65.9 %	
No	6644	51.9 %	523	35.9 %	942	43.4 %	117	31.0 %	
Not sure	387	3.0 %	48	3.3 %	56	2.6 %	12	3.2 %	
Taking steps to prevent osteoporosis	919	7.2 %	176	12.1 %	561	25.9 %	124	32.8 %	<0.0001
Steps taken to prevent osteoporosis									
Take calcium	571	62.1 %	123	69.9 %	362	64.5 %	77	62.1 %	0.2418
Exercise regularly	479	52.1 %	110	62.5 %	298	53.1 %	65	52.4 %	0.0888
Drink or eat dairy products (e.g., milk, yogurt)	755	82.2 %	146	83.0 %	476	84.8 %	99	79.8 %	0.4428
Take a prescription medication	97	10.6 %	33	18.8 %	114	20.3 %	43	34.7 %	<0.0001
Take vitamin D	220	23.9 %	56	31.8 %	144	25.7 %	43	34.7 %	0.0185
Take steps but none of the above	6	0.7 %	1	0.6 %	0	0.0 %	1	0.8 %	0.2849
Family history of osteoporosis	371	2.9 %	49	3.4 %	338	15.6 %	65	17.2 %	<0.0001

*BMI* body mass index, *ER* emergency room, *CCI* Charlson Comorbidity Index

^a^ *p* value according to one-way ANOVA

^b^ *p* value according to Pearson’s chi-square

Those who felt at risk for developing osteoporosis in the future were more likely to indicate that they were taking steps to prevent osteoporosis, with 33 % of those with a prior fracture and 26 % of those without a prior fracture taking preventative steps, compared to 12 and 7 % of those not feeling at risk with and without a fracture, respectively. The specific preventative steps taken were largely similar across risk groups and were most often the consumption of dairy products, reported by 80 to 85 % of those who reported taking preventative steps. BMD scanning was most common among those feeling at risk for osteoporosis and varied from 45 % among those not feeling at risk and without fracture to 66 % among those feeling at risk with a fracture. Those without a fracture who did not feel at risk were least likely to have visited a physician in the prior 6 months, followed by those who did not feel at risk but had a fracture since age 50. Having an emergency visit in the prior 6 months was approximately twice as likely among those who experienced a fracture since age 50 as those who did not have fractures since age 50, and a similar pattern was seen with having a hospital visit.

The relationships between previously identified risks for fracture and perceived risk of developing osteoporosis were generally weak, with some exceptions. Family history was rare among those not feeling at risk, at approximately 3 % for both groups, and approximately five times more common among those feeling at risk for developing osteoporosis at 16 and 17 % for those who felt at risk for developing the disease. Back pain was also associated with perceived risk, up to twice as common among those who felt at risk. In contrast, other predictors of risk included in FRAX were not strongly associated with perceived risk, including current smoking, daily alcohol use, and use of oral glucocorticoids.

Bivariate comparisons of health outcomes demonstrated that health status, work, and activity impairment were generally best among the group not feeling at risk and without fractures, though differences were relatively small (Table 2). Likewise, this group had the fewest physician visits and the lowest number of hospitalizations in the prior 6 months.

**Table 2:** Unadjusted health outcomes among women in Japan age 50 and older by perceived risk of osteoporosis and fracture history

	Not feeling at risk, no fractures		Not feeling at risk, fracture		Feeling at risk, no fractures		Feeling at risk, fractures		
	Mean	SD	Mean	SD	Mean	SD	Mean	SD	*p* value
MCS	49.49	9.40	49.54	10.02	47.00	10.25	48.13	9.58	<0.0001
PCS	50.48	6.89	48.54	8.10	48.97	7.36	47.91	7.97	<0.0001
SF-6D	0.779	0.127	0.766	0.137	0.739	0.126	0.745	0.127	<0.0001
Absenteeism (%)	2.6 %	11.9 %	5.4 %	17.4 %	2.3 %	9.8 %	4.4 %	14.3 %	0.0001
Presenteeism (%)	12.3 %	19.0 %	13.0 %	20.6 %	15.0 %	19.9 %	17.1 %	23.3 %	0.0004
Overall work impairment (%)	14.0 %	21.6 %	16.3 %	25.4 %	16.2 %	21.7 %	18.1 %	25.8 %	0.0053
Activity impairment (%)	16.5 %	21.5 %	21.1 %	25.3 %	21.3 %	23.9 %	22.5 %	23.9 %	<0.0001
Physician visits	5.48	8.09	8.23	9.92	7.09	9.81	9.08	9.94	<0.0001
ER visits	0.09	1.18	0.16	1.03	0.07	0.73	0.09	0.58	0.0891
Hospitalizations	0.41	3.67	1.24	7.68	0.46	3.68	0.51	2.82	<0.0001

Higher scores on MCS and PCS indicate better health status. *p* values are from one-way ANOVA

*MCS* mental component summary, *PCS* physical component summary, *ER* emergency room

Generalized linear regression analysis of health outcomes revealed a generally consistent picture (Table 3). The group not feeling at risk and without fractures had significantly higher MCS and PCS scores than the three other groups, though none reached the 3-point threshold for minimally important difference. Only a few small differences in work productivity impairment were observed, with higher absenteeism among those not feeling at risk with a fracture relative to the reference group and higher presenteeism among those feeling at risk. Impairment to non-work activities (19 %) was significantly lower than those of the other three groups (24–25 %). The reference group also had fewer physician visits than all the other groups, fewer ER visits than those not feeling at risk but with a fracture, and fewer hospitalizations than that group as well.

**Table 3:** Regression-adjusted mean health outcomes by perceived risk of osteoporosis and fracture history

	Not feeling at risk, no fracture	Not feeling at risk, fracture	Feeling at risk, no fracture	Feeling at risk, fracture
	Mean (SE)	Mean (SE)	Mean (SE)	Mean (SE)
Health-related quality of life				
MCS	48.1 (0.2)	47.2*** (0.3)	45.9*** (0.3)	45.9*** (0.5)
PCS	48.5 (0.1)	47.1*** (0.2)	47.0*** (0.2)	46.3*** (0.4)
SF-6D	0.75 (0.00)	0.73*** (0.00)	0.72*** (0.00)	0.71*** (0.01)
Work and activity impairment				
Absenteeism (%)	3.5 % (0.5)	6.7 %** (1.8)	2.9 % (0.6)	7.4 % (3.6)
Presenteeism (%)	13.7 % (0.7)	14.9 % (1.3)	15.7 %* (1.1)	20.7 %** (3.3)
Overall work impairment (%)	15.7 % (0.8)	18.3 % (1.6)	17.4 % (1.2)	22.2 %* (3.5)
Activity impairment (%)	19.0 % (0.5)	23.6 %*** (1.0)	24.0 %*** (0.9)	25.3 %*** (1.8)
Healthcare use (6 months)				
Physician visits	6.4 (0.2)	8.4*** (0.4)	8.2*** (0.3)	9.9*** (0.8)
ER visits	0.08 (0.02)	0.13* (0.04)	0.06 (0.02)	0.07 (0.04)
Hospitalizations	0.42 (0.07)	1.04*** (0.28)	0.44 (0.11)	0.51 (0.24)

All models adjusted for age, university education, smoking status, exercise, daily alcohol use, household income, BMI category, marital status, and the CCI. Mean and SE presented at the mean of the covariates. Higher scores on MCS and PCS indicate better health status

*MCS* mental component summary, *PCS* physical component summary, *ER* emergency room

Significance is relative to group not feeling at risk and without fractures; *p < 0.05, **p < 0.01, ***p < 0.001

The results of the logistic regression of feeling at risk for developing osteoporosis in the future demonstrated that some variables usually considered outcomes were associated with feeling at risk for osteoporosis (Table 4). Lower MCS and PCS scores were associated with higher adjusted odds of feeling at risk for developing osteoporosis in the future, as was visiting a physician at least once in the past 6 months. Being hospitalized in the prior 6 months was associated with slightly lower adjusted odds of feeling at risk. Osteoporosis-related factors associated with feeling at risk included prior fracture since age 50, back pain, and having completed menopause. Having a BMD scan was associated with higher odds of feeling at risk than not having a BMD scan. Family history of osteoporosis was particularly closely associated with feeling at risk for developing osteoporosis, while use of glucocorticoids, alcohol use, and smoking were not associated with feeling at risk. BMI category was also associated with feeling at risk for developing osteoporosis, with those underweight more likely to feel at risk than those of normal weight and those overweight or obese less likely to feel at risk. Finally, demographics were also related to feeling at risk, with younger age and declining to answer income associated with lower adjusted odds of feeling at risk, while college education was associated with higher odds of feeling at risk.

**Table 4:** Association between respondent characteristics and feeling at risk of developing osteoporosis

Factor	Odds ratio	95 % confidence limits		*p* value
		Lower	Upper	
Age (5-year interval)	0.95	0.91	0.99	0.008
CCI	1.04	0.94	1.14	0.4734
Fracture (since age 50)	1.39	1.22	1.58	<0.0001
PCS (5-point interval)	0.89	0.85	0.92	<0.0001
MCS (5-point interval)	0.90	0.88	0.93	<0.0001
Activity impairment (5 % interval)	0.99	0.98	1.01	0.3341
What is your marital status?				
Married/living with partner	Reference category			
Never married	1.08	0.95	1.22	0.2609
Widowed	1.00	0.84	1.19	0.9712
Employed	0.96	0.86	1.06	0.3681
Completed 4-year college	1.12	1.01	1.23	0.0354
Annual household income				
Below ¥5,000,000	Reference category			
¥5,000,000 or above	1.09	0.98	1.20	0.1154
Decline to answer	0.61	0.52	0.72	<0.0001
BMI category				
BMI: Underweight	1.32	1.15	1.50	<0.0001
Normal	Reference category			
Overweight	0.62	0.53	0.72	<0.0001
Obese	0.46	0.30	0.70	0.0003
Decline to answer	0.53	0.39	0.72	<0.0001
Currently smokes	1.11	0.97	1.26	0.1239
Currently exercises	1.05	0.96	1.15	0.3286
Daily alcohol use	0.92	0.80	1.06	0.2310
Back pain	1.33	1.11	1.58	0.0015
On glucocorticoids	1.09	0.84	1.43	0.5125
Completed menopause	1.25	1.12	1.39	<0.0001
Visited physician (in the prior 6 months)	1.41	1.27	1.58	<0.0001
Visited ER (in the prior 6 months)	0.85	0.66	1.09	0.1927
Visited hospital (in the prior 6 months)	0.79	0.63	1.00	0.0479
Have you ever had a bone mass density test/scan?				
Yes	1.32	1.20	1.44	<0.0001
No	Reference category			
Not sure	1.10	0.84	1.45	0.4780
Family history of osteoporosis	5.52	4.75	6.41	<0.0001

*MCS* mental component summary, *PCS* physical component summary, *ER* emergency room

## Discussion

Osteoporosis is often termed a silent disease because individuals do not realize they have developed the condition, and the disease progresses without symptoms until the individual experiences a clinical fracture. As osteoporotic spine fractures often go undetected, individuals may even suffer multiple osteoporotic fractures prior to diagnosis [22]. The current analysis demonstrated that women age 50 and older in Japan generally do not feel at risk for developing osteoporosis in the future, though many will. Though individuals reporting a physician diagnosis of osteoporosis were excluded from the present study, it is possible that some of the respondents included in the present study in fact already have osteoporosis and are simply unaware of the weakened state of their bones. Indeed, while the majority of those who had experienced a fracture since age 50 indicated having a BMD scan, a substantial minority—approximately one in three—indicated they had never had a BMD scan, which is an integral part of the diagnostic procedure according to Japanese guidelines [16]. Thus, many of the respondents had not had the opportunity to be diagnosed with osteoporosis. Few indicated they were actively taking steps to prevent osteoporosis: slightly more than one in every four who felt at risk for developing it in the future and only about one in every ten among those who did not feel at risk. Feelings of risk themselves were most associated with family history of osteoporosis, though rates of BMD scanning and having visited a physician in the prior 6 months also differed across the groups and seemed to vary primarily in relation to perceived risk, suggesting that feeling at risk may be due to a mixture of family history and engagement with healthcare providers. The regression analysis directly assessing which factors were associated with perceived risk also indicated a strong relationship with family history and weaker relationships with recently seeing a physician, having a previous fracture since age 50, and lower mental and physical quality of life, among other factors.

Though previous fracture is widely understood to be an important predictor of future fracture [14], the relationship between fracture history and perceived risk of osteoporosis was quite small. Though there were differences across groups, no clear relationship emerged between perceived risk of osteoporosis and other predictors of fracture aside from family history. Smoking, alcohol use, and use of glucocorticoid medication were quite similar across the groups, indicating that women are either unaware of the relationship these have with risk of osteoporotic fractures or the knowledge does not have enough impact to affect their perceived risk for developing osteoporosis. The pattern of results was similar when using logistic regression, with none of those behavioral risk factors significantly associated with feeling at risk.

The pattern of results in regression analysis demonstrated that perceived risk and fracture history are related to outcomes, but these relationships are modest in size among those who do not have osteoporosis. Those with fractures among women age 50 and older in Japan have worse health outcomes than those who do not experience fractures, which seems to be clearest in the PCS and SF-6D scores, while lower PCS scores themselves are also associated with feeling at risk for developing osteoporosis. Analyses of work productivity impairment had limited power to detect differences, as only a minority of the sample was employed, and those who had not had a fracture were more likely to be working.

The current study has several limitations which should be considered. All information, including presence or absence of fractures, was assessed through self-report and could not be confirmed. The measure of perceived risk was a part of a checklist rather than a more sensitive measure, and the wording indicated the future in general rather than a designated time frame. This makes the comparability of the reported risk perception and epidemiological risk factors less clear. Use of the FRAX score to indicate risk of fracture rather than individual predictors would also have allowed for a stronger comparison. Likewise, it is worth noting that the perceived risk included in the NHWS was risk of developing osteoporosis, not perceived risk of a fracture. To the extent that these risks are perceived differently, the risk of fracture would be a more appropriate perception to compare with risk factors for fracture. An interesting direction for future research would be to incorporate a history of falls or risk factors for falls. Falling is often the cause of osteoporotic fractures, but this information regarding falls was not included in the NHWS [23]. The modest response rate of the survey may have resulted in some self-selection bias to the results, though the current survey indicated the prevalence of self-reported fracture in the current survey among menopausal women was similar to that previously reported in the literature (16 % [24]). The survey respondents were sourced through an opt-in Internet survey panel and therefore may differ in important ways relative to the population as a whole. By definition, all were Internet users, and though Japan has one of the highest rates of Internet use in the world (approximately three of every four individuals accessed the Internet in 2007, the year prior to the first survey included here), Internet use was less common among older adults [25]. Therefore, reliance on an Internet-based panel may have biased estimates, such as the mean age of the respondents and the proportion of women feeling at risk, having experienced a fracture, or who had received a bone mineral density scan, though it seems less likely that this would impact the relationships observed between variables. The accuracy of the lifetime rate of BMD scanning is particularly hard to assess, as we are not aware of any published population-based studies of lifetime prevalence of these scans among women aged 50 and older in Japan or elsewhere. This rate may be an overestimate, as the rate reported here is higher than reported rates of BMD scanning in other studies reporting BMD testing rates in large samples outside Japan, though the rates reported elsewhere are not lifetime rates [26–28]. The number may also reflect the availability of bone measurement at locations other than hospitals or medical offices in Japan, as calcaneal qualitative ultrasound (QUS) is sometimes made available in public places during health promotion events to raise awareness of osteoporosis and fracture risk. Some women may have mistakenly indicated having had a BMD scan when in fact they have undergone QUS measurement. Finally, because of the cross-sectional and non-interventional nature of the study, the associations observed in the study should be considered correlational rather than indicating causal relationships.

In conclusion, the present study adds to the evidence that risk for osteoporosis is not well understood by the segment of the Japanese population most at risk for fractures, women age 50 and older. Those at risk for osteoporosis do not realize they are at risk, and few risk factors are strongly associated with perceived risk aside from family history. Furthermore, even some of those who do feel at risk are not necessarily taking steps to protect themselves against bone loss and future fractures. The lack of awareness and prevention is troubling considering the high personal, social, and economic cost of osteoporotic fractures, especially in the context of an increasingly aged populace such as Japan. These results suggest that in Japan, as elsewhere in Asia, continued efforts are needed to raise awareness of osteoporosis risk and available preventative measures.
